# ‘he’s not just a dog… he’s something bigger… my family.’ A Qualitative Study on Dog Ownership and Emotional Well-Being

**DOI:** 10.3390/healthcare13212666

**Published:** 2025-10-22

**Authors:** Eirini Stamataki, Panagiota Tragantzopoulou

**Affiliations:** 1 School of Psychology, University of Derby, Derby DE22 1GB, UK; eiri.stamataki@mc-class.gr; 2 School of Social Sciences, University of Westminster, 115 New Cavendish St., London W1W 6UW, UK

**Keywords:** dog adoption, dog ownership, companionship, emotional well-being, loneliness, thematic analysis

## Abstract

**Background/Objectives**: Dogs are widely regarded as reliable sources of companionship and emotional support. In many instances, they are not merely considered pets, but valued as integral members of the family who significantly influence their caregivers’ emotional and psychological health. Within this framework, this research examines how dog ownership through adoption may serve as both a protective and empowering factor against feelings of loneliness, while also fostering emotional resilience and a renewed sense of purpose in everyday life. **Methods**: Employing a qualitative research design, this study involved ten Greek participants, five women and five men, aged between 26 and 72, all of whom were the primary caregivers of their dogs. Data were collected through semi-structured interviews aimed at eliciting rich, in-depth personal narratives. Thematic analysis was used to identify recurring emotional patterns and explore the meanings embedded in participants’ accounts. **Results**: The findings revealed that the human–dog bond functions as a stable emotional anchor, promoting non-judgmental connection and emotional security. Participants reported experiencing greater emotional expression, enhanced social engagement, and improved psychological balance. **Conclusions**: Overall, the results demonstrate how dog ownership through adoption may act as a protective factor against loneliness while fostering resilience and emotional balance, pointing to the broader mental health benefits of nurturing human–animal bonds.

## 1. Introduction

Pet ownership is a longstanding, cross-cultural practice deeply embedded in human history and widely associated with psychosocial benefits [1]. In contemporary Western societies, pets, especially dogs, have evolved beyond utilitarian roles to become emotionally significant companions [2]. In countries like the United States, Canada, and Australia, more than two-thirds of households own a pet, with dogs and cats being the most common [3,4,5]. Dogs, in particular, are often viewed as reliable sources of emotional support and companionship, fostering reciprocal human–animal bonds [6,7]. This emotional closeness has led to pets being considered full-fledged family members, influencing their owners’ emotional and psychological well-being [8,9,10,11]. The emotional benefits of such relationships are closely tied to fundamental human needs for social connection and belonging [12]. Particularly in the context of growing loneliness—a phenomenon increasingly described as a modern epidemic in individualistic societies—dogs have become salient as both emotional and social support resources [13,14].

### 1.1. Loneliness and Emotional Well-Being

Loneliness refers to the subjective perception of unsatisfying social connection, distinct from objective social isolation [9,15]. This state is consistently associated with negative health outcomes, including depression, anxiety, cardiovascular issues, and early mortality [16,17]. Companion animals—especially dogs—have been proposed as potential buffers against loneliness by offering routine, emotional connection, and daily interaction [1,18].

Emotional well-being includes constructs such as positive affect, emotional regulation, resilience, and life satisfaction [19,20]. Dogs support these through dynamic and responsive companionship, marked by physical closeness and non-verbal attunement [21,22]. These relationships can enhance psychological safety and emotional expression, especially for individuals with limited human support [23]. In this way, dogs serve not just as passive companions but as emotional stabilizers through structured care routines that provide agency and purpose [24,25]. Moreover, dogs can attenuate physiological stress responses and increase feelings of calmness, tenderness, and affection, contributing to an improved emotional state even in the absence of human companionship [26,27]. For individuals facing chronic loneliness or social disconnection, the human–dog bond can strengthen self-worth and instill feelings of being valued and loved—critical factors for mental health [12].

### 1.2. Social Effects of Dog Adoption on Loneliness and Social Relationships

Beyond emotional comfort, dogs serve as catalysts for social interaction, acting as “social facilitators” who lower barriers to engagement and promote interpersonal connection [28,29]. Dog walking, in particular, has been linked to increased community engagement, informal conversations, and a sense of neighborhood belonging [2]. These encounters often progress from casual interactions to meaningful social relationships, helping mitigate social isolation [30]. While the presence of a dog may not expand social networks quantitatively [31,32], the quality of social engagement it promotes—through visibility, shared activities, and reduced social anxiety—is of greater psychological significance [33]. This is especially relevant for those facing marginalization or life transitions, where a dog’s presence can serve as a bridge to social reintegration.

### 1.3. Psychological Effects of Dog Ownership on Emotional Well-Being and Loneliness

Dog ownership specifically has been increasingly recognized as a source of emotional and psychological resilience. Dogs are adept at reading and responding to human emotional cues, offering emotional stability and helping to reduce stress, anxiety, and depressive symptoms—particularly during periods of emotional strain [34,35,36]. The caregiving routine—feeding, walking, grooming—offers structure and purpose, reinforcing self-efficacy and mental balance [12,24]. During the COVID-19 pandemic, for instance, dog owners reported that their pets offered distraction, emotional grounding, and relief from loneliness and anxiety [37]. These findings underscore the dog–human bond as a form of emotional anchoring that can supplement or substitute for traditional social support [38]. Emotional attachment to dogs has also been shown to enhance self-esteem and self-worth, particularly during adversity [25,39].

However, not all studies confirm a direct link between pet attachment and improved psychological outcomes. For example, a study of Greek male pet owners found that while higher pet attachment predicted greater perceived social support, it did not significantly correlate with reduced loneliness or improved psychological well-being [11]. These results may reflect limitations such as the cross-sectional design, self-report bias, and the unique social context of the pandemic, which may have influenced emotional reliance on pets without fully mitigating broader psychological burdens.

### 1.4. Companion Animals in Greece: Strays and Ownership Trends

Greece faces one of the largest populations of stray companion animals in the world, with estimates suggesting that around three million dogs and cats currently live on the streets [40,41]. Projections from Aristotle University of Thessaloniki indicate that this number may rise to nearly four million in the coming years [42]. At the same time, pet ownership trends have changed considerably over the last two decades. A growing awareness of animal welfare has coincided with more positive social attitudes toward companion animals, contributing to an increase in ownership [43]. Current surveys show that approximately 43% of the Greek population own at least one companion animal. Of these, two-thirds (66.1%) report having a dog—about 655,000 animals—while around 42% keep cats, equating to roughly 606,000 [43,44]. Ownership patterns also vary across age groups, with younger Greeks more likely to own pets compared to retirees, who represent the lowest proportion of companion animal guardians [43,44]. Despite the growing interest in companion animals, there is a notable gap in research on the practice of adopting animals in Greece, particularly regarding the experiences of adopters and the adjustment process. International studies have highlighted that adoption is a distinct process involving a transitional “adjustment period” during which both the animal and the owner adapt to new routines and relationships [45]. This period can be marked by behavioral changes in dogs, including play, tail wagging, and learning routines, and is influenced by factors such as prior experiences and the dog’s needs. These factors directly affect owner satisfaction, perceptions of the adoption experience, and long-term success [46]. Research also shows that adopters often make specific decisions based on a dog’s behavior, size, and temperament, aiming to ensure compatibility and avoid relinquishment [46]. However, such studies remain absent in Greece, leaving unanswered questions about how the process of adoption unfolds in this cultural context.

### 1.5. The Process of Dog Acquisition and Emotional Implications

While much research has examined the outcomes of pet ownership, less attention has been given to how individuals come to acquire their pets in the first place. Qualitative research suggests that dog acquisition can be either intentional—driven by prior planning and personal preferences—or unplanned, often occurring through chance encounters, social networks, or emotional impulse [47,48,49]. In the latter cases, people frequently report an immediate emotional connection to the animal and make adoption decisions without extensive research or preparation. These findings highlight how the process of acquisition itself—particularly when unplanned—can shape the nature of the human–animal bond and may carry implications for both emotional outcomes and responsible pet ownership.

Although scientific interest in the relationship between dog ownership and mental health has grown in recent years, the literature remains overwhelmingly quantitative, relying on correlational findings to assess links between pet ownership and psychological outcomes. While these studies offer important insights, they often fail to capture the complex, subjective nature of individuals’ emotional relationships with their dogs [50]. Constructs such as loneliness, emotional resilience, and well-being are deeply experiential and context-dependent, making them difficult to fully understand through standardized scales alone. As a result, much of the existing literature risks oversimplifying how emotional benefits are experienced, negotiated, and interpreted by dog owners in their everyday lives.

While some qualitative studies have examined adjacent topics—such as adopters’ concerns before acquiring a dog [51], the reasoning behind dog acquisition [48], and the adjustment period following rehoming [52,53]—these inquiries have primarily focused on decision-making processes, animal welfare, or the dog’s behavior and integration into the household. These are valuable contributions, yet they largely overlook the emotional impact of adoption on the owners themselves. The specific ways in which dog companionship may influence emotional resilience, social connectedness, or mental equilibrium post-adoption remain underexplored. No existing qualitative research has systematically addressed how adult dog owners experience and interpret these emotional shifts in their day-to-day lives, leaving a significant gap in understanding the human side of the adoption dynamic.

This study seeks to address this limitation by adopting a qualitative approach to explore the emotional impact of dog ownership, with particular attention to how companionship may foster emotional resilience, reduce feelings of isolation, and support psychological balance. Through in-depth, person-centered inquiry, the research aims to provide a more textured and context-sensitive understanding of how dog ownership intersects with the inner emotional world of adult adopters, advancing knowledge of its role in psychosocial functioning beyond what quantitative methodologies alone can reveal.

## 2. Materials and Methods

### 2.1. Research Design

This study employed a qualitative research design to explore how individuals who adopted a dog experienced changes in loneliness and emotional well-being. A qualitative approach was deemed appropriate to capture the subjective, contextual, and nuanced dimensions of participants’ lived experiences—elements often overlooked in quantitative paradigms [54]. The study was epistemologically grounded in interpretivism, which posits that reality is socially constructed and meaning is derived through individuals’ subjective interpretations of their experiences [55]. Within this framework, thematic analysis, as outlined by Braun and Clarke, was selected as the guiding methodological approach. Thematic analysis provided the structure for the entire study, from framing the research questions to shaping the process of data collection and interpretation. This integration of interpretivism and thematic analysis positioned the research to prioritize depth over breadth, focusing on the richness and authenticity of individual narratives rather than on the generation of generalizable findings. By explicitly aligning the study’s design with the chosen method of analysis, the approach ensured methodological coherence and transparency. This interpretivist stance informed not only the analytic choices but also the interactive nature of data collection, where meaning was understood as co-constructed between researcher and participant.

### 2.2. Participants

The sample comprised ten adult participants (five men and five women), aged between 26 and 72 years, all of whom were Greek nationals and had adopted at least one dog (see Table 1). Participants were recruited through purposive convenience sampling, initiated within the researcher’s extended social network and expanded via targeted open calls on social media and email lists. This approach provided a pragmatic entry point into a population where formal registries of dog adopters do not exist. To address potential bias associated with proximity to the researcher, reflexive practices were applied throughout recruitment and analysis, including maintaining analytic memos that documented researcher assumptions and reactions to participants’ accounts. This reflexivity supported transparency in interpreting participants’ narratives and minimized undue influence of familiarity on data interpretation. Eligibility criteria required participants to be over 18 years of age and to have adopted a dog at least three months prior to the interview to ensure sufficient reflection time. No maximum timeframe was applied, which allowed the inclusion of both relatively recent and long-term owners. While this broadened the diversity of experiences captured, it also introduced variability between participants who were reflecting on the immediate aftermath of adoption and those describing more established, long-term ownership. Individuals were excluded if they were under 18, currently undergoing active psychological treatment, or had a diagnosed severe mental health disorder, to safeguard participant well-being and maintain data integrity. The sample size was appropriate for the aims of a small-scale qualitative inquiry, providing analytic depth while ensuring data manageability in alignment with the interpretivist framework [56]. In line with ethical guidelines and to safeguard participant confidentiality, all participants are referred to using code names in the tables and data excerpts presented throughout the study.

### 2.3. Data Collection

Data were collected through in-person, semi-structured interviews conducted in Greek—the participants’ native language—to ensure emotional authenticity and depth of expression [57]. The data collection process took part between November and December 2024 and each interview lasted between 40 to 50 min. Interviews were conducted in a private setting within the first author’s university department, providing a comfortable and confidential environment. Interested individuals responded to an open call for participation. They received a detailed information sheet outlining the study’s aims, procedures, and ethical safeguards and were required to provide written informed consent prior to participation.

An interview guide was developed collaboratively by the first and second authors, drawing on existing literature related to human–animal interactions, emotional well-being, and pet adoption experiences. It consisted of 20 open-ended questions designed to elicit rich personal narratives concerning participants’ emotional states, social adjustments, and coping mechanisms following dog adoption (e.g., “Do you notice any changes in your mood after spending time with your dog?”, “Has your dog helped you manage any negative emotions?”, “Do you feel your social life has changed since adopting your dog?”). Along with the guide, prompts such as “Can you give an example?” and “How did that make you feel?” were utilized throughout the interviews to encourage elaboration and facilitate depth.

In line with the interpretivist stance, the study assumed that meaning is co-constructed between researcher and participant during the interview process. Accordingly, the interview guide was designed to elicit reflective, meaning-oriented narratives rather than factual descriptions. The first researcher maintained a reflexive stance throughout data collection and analysis, acknowledging their own interpretive position and prior experience as a dog owner, and how this shaped both engagement with participants and meaning-making during coding.

All interviews were audio-recorded with participants’ consent and transcribed verbatim in Greek. The transcripts were then translated into English for analysis to facilitate integration of the findings into the international literature. Particular care was taken to preserve participants’ original meanings and emotional tone during translation, with back-checking of key excerpts against the original recordings to ensure accuracy. Translation was therefore conceptualized as an interpretive act rather than a mechanical conversion of language. The research team approached it reflexively, discussing how idiomatic expressions and culturally embedded meanings might shift in translation. This process enhanced the dependability of the data and ensured that the analytical claims remained grounded in participants’ intended meanings. This approach aligns with the stance that views translation as a crucial stage of meaning-making rather than a technical step in multilingual qualitative research. Field notes were also taken to document contextual observations and non-verbal expressions. Upon conclusion of the interview, participants were provided with a debriefing form and reminded of their right to withdraw their data within two weeks, using a pseudonym of their choice. No participant opted to withdraw from the study, and all identifiable information was omitted to ensure confidentiality.

### 2.4. Data Analysis

Data were analyzed using Braun and Clarke’s six-phase framework for thematic analysis [58,59,60], chosen for its systematic yet flexible approach and consistency with the study’s interpretivist orientation. This method allowed for an in-depth exploration of the meanings participants attributed to their experiences while acknowledging the role of researcher subjectivity in theme development.

Guided by our interpretivist framework, we approached the data as co-constructed narratives reflecting both participants’ and researchers’ interpretations of experience. Analysis began with repeated reading of the English transcripts alongside listening to the original Greek recordings to maintain linguistic nuance and cultural context. Initial coding was conducted independently by both authors using an inductive, data-driven approach. No a priori codes were imposed; instead, both semantic (explicit) and latent (underlying) meanings were captured to ensure a comprehensive representation of participants’ perspectives. Following this phase, the researchers compared their independent codebooks. Through iterative discussion, codes were refined, merged, or re-labeled, and grouped into preliminary categories. Broader themes were then collaboratively developed, defined, and named to reflect their content. While inter-rater reliability was not quantified, analytical rigor was ensured through reflexive dialogue, consensus-building, and the documentation of all coding and thematic decisions. This approach aligns with the principles of reflexive thematic analysis, which emphasizes the active role of the researcher and prioritizes transparency and interpretive depth over statistical agreement. The final themes were defined and named to accurately reflect their content, and supported with direct quotations from participants to illustrate key ideas and preserve the authenticity of their voices. 

Analytical rigor and trustworthiness were established through multiple strategies. Credibility was enhanced by iterative reading, detailed documentation of analytic decisions, and ongoing reflexive discussions between authors to surface assumptions and alternative interpretations. Dependability was supported by maintaining an audit trail of coding iterations and theme development. Confirmability was ensured by anchoring all claims in verbatim excerpts, and transferability by providing rich contextual detail about participants and the research setting. This reflexive thematic analysis approach follows Braun and Clarke’s later interpretivist model, which emphasizes subjectivity and meaning-making rather than inter-coder reliability as the standard of quality. Accordingly, consensus discussions replaced statistical measures of agreement, focusing instead on achieving depth and coherence in theme development.

### 2.5. Ethical Considerations

The study received ethical approval from the first author’s university ethics committee and was conducted in accordance with institutional guidelines for research involving human participants. Ethical procedures were followed throughout all stages of the study to ensure the rights, dignity, and welfare of participants were protected. All participants received a detailed information sheet outlining the purpose of the research, procedures, and their rights, including the voluntary nature of participation. Written informed consent was obtained prior to data collection.

To protect participants’ privacy, anonymity was ensured through pseudonymization, and no personally identifiable information was collected. Data were securely stored on encrypted, password-protected devices accessible only to the authors. Participants were reminded of their right to withdraw at any point during the process, without consequence, and were given two weeks after the interview to request withdrawal of their data. Reflexivity was embedded as an ethical practice throughout the study, recognizing the researcher’s dual role as both listener and interpreter of participants’ emotionally charged experiences.

## 3. Results

Two main themes were identified, each comprising two sub-themes (Table 2). These findings illustrate how participants described their relationship with their dog and its impact on loneliness and emotional well-being. Verbatim excerpts are included to preserve the authenticity of participants’ voices.

### 3.1. Alleviating Loneliness Through Companionship

#### 3.1.1. Emotional Connection with the Dog

One of the central themes that emerged from participants’ narratives was the depth of the emotional bond they described with their dog. For many, this connection was not limited to notions of ownership but carried personal and relational significance, with dogs often becoming integrated into participants’ daily lives and, in some cases, their family routines. Participants frequently described their dog as a consistent source of comfort and stability, particularly during periods of loneliness, loss, or emotional vulnerability.

One participant, for instance, described a period of mourning in which his dog became his only source of comfort. As he characteristically stated, “*He was my only consolation during that time*”. This was not a temporary relief, but rather the beginning of a strong emotional bond, as he later added, “*A new family was being formed*”. The dog was not simply a response to emotional distress but gradually evolved into a central pillar of his new family reality.

Similar accounts were shared by other participants, who referred to their dogs as family members, “*he is a part of my family*”, “*she is part of my soul… my child*”, and “*he’s not just a dog… he’s something bigger… A is my family*”. These statements reveal how the relationship participants had built with their dogs transcended everyday cohabitation, becoming a companionship rooted in emotional safety, shared experiences, and deep connection.

The dog’s presence was often described as comforting and reassuring, with many participants referring to small actions, glances, or physical contact that held significant weight in their psychological stability. Especially during challenging and stressful periods, the dog seemed to take on a supportive, at times even therapeutic, role. More specifically, one of the participants recounted turning to her dog during a moment of anxiety while preparing for a public presentation, “*I told her*, *‘Come on*, *N*, *let’s say it one more time*,*’ and she sat on her hind legs and looked me in the eyes… and that really helped me*”, she said. This interaction illustrates the dog’s unique ability to provide emotional relief simply through its presence, functioning as a steady emotional companion in times of stress.

In other cases, the dog was described as an “*anchor*” or a “*balm*” during emotionally turbulent times, highlighting the life-sustaining dimension of this relationship, a bond not merely supportive, but vital to the individual’s emotional survival. As one participant shared, “*During COVID*, *which was a really tough time… and I had just gone through a breakup as well*, *so you can imagine… if S wasn’t in my life*, *I honestly can’t imagine how much worse things would’ve been… without S*, *I’d clearly have been in a state of depression*”.

The strength of this bond is also reflected in more concise yet deeply emotional expressions, such as “*I wouldn’t trade what we have for anything*”, “*It’s the purest bond I have in my life*”, and “*I can’t imagine my life without M*”. These declarations capture the intensity of the emotional intimacy, devotion, and dependence that had been cultivated. What emerges is a lived, empowering connection that goes far beyond the traditional pet-owner relationship, positioning the dog as a constant and meaningful psych-emotional presence in the participants’ lives. While the tone and depth of these expressions varied slightly across individuals, the underlying experience of emotional closeness and inner resilience appeared to be shared among all participants.

#### 3.1.2. Transformation of Well-Being

Another important aspect that emerged from the participants’ narratives was the noticeable change in their emotional state following the adoption of their dog. These changes mainly concerned an increase in positive emotions, a reduction in negative ones, and a renewed sense of purpose and motivation in their daily lives.

Some participants emphasised the positive psychological impact of physical contact with their dog. Statements such as “*when I come home and we cuddle… it’s like she gives me energy I didn’t have before*”, “*it’s incredible… how just touching him changes my whole mood*”, and “*when we play*, *he transmits his joy to me… I feel happy with his happiness every single time*”, reflect the experience of emotional renewal that stems from physical closeness and the animal’s emotional responsiveness. Participants described their dog not only as a source of comfort but also as a source of energy and vitality that brings emotional freshness and positivity to everyday life.

Others focused on the transformative effect the dog had on their daily routine, particularly on how they started their day. One participant, for instance, noted, “*I used to wake up grumpy… but now with M… I’m happy within seconds*”, illustrating the immediate emotional uplift the dog’s presence provided. This immediate impact was also expressed by another participant who shared, “*I feel that Z brought life*, *meaning*, *and laughter back into my days*”, suggesting that the dog helped transform mundane daily moments into experiences of joy and emotional fulfilment.

At the same time, participants placed particular emphasis on the alleviation of negative emotions, describing their dog’s presence as calming, comforting, and emotionally protective, easing the psychological burden of daily life. Specific examples include, “*M’s presence… reduces the sadness I feel at various points during the day*”, “*I stopped feeling lonely and he magically took away all my worries*”, and “*I used to feel deeply alone*, *but when I held M in my arms*, *I felt relieved*”. These narratives illustrate how the relationship with the dog functioned as a mechanism of emotional release and protection, offering a safe and familiar space in which participants could relax and feel less vulnerable and alone.

Sadness, often described as persistent or overwhelming, was perceived as significantly diminished through the dog’s presence. One of the participants stated, “*I no longer feel like I’m sinking into sadness. Z helps me push it away…*”, expressing not only a shift in emotional experience but also a sense of empowerment, as the dog was seen not just as a comforting presence but as an active support in managing emotional pain. Similarly, another participant noted, “*He helps me unwind after stressful situations… even when I’m upset*, *he helps me forget somehow*”. In this account, the dog is portrayed as a calming agent and a means of emotional detachment, facilitating relief from distress and creating space for inner calm and emotional recovery.

Beyond the emotional benefits, many participants described how living with a dog offered them a renewed sense of purpose, an everyday motivation that helped them stay active and regain a sense of rhythm in their lives. Two participants, reflecting on periods of bereavement, shared, “*I wouldn’t have made it through without K… he was the reason I smiled during the day… even when I didn’t feel like doing anything… I’d do it for K… I had to take care of him and take him out for a walk*”, and “*He keeps me going… gives me a reason to get out of the house*”. These narratives show how the responsibility of caring for a dog became a psychological anchor, particularly during moments of emotional breakdown, turning routine care into a daily reminder of life and re-engagement with the world.

These excerpts clearly illustrate the transformation of daily routine into a meaningful experience. Through their basic needs, dogs reintroduce structure, purpose, and a steady sense of presence into their owners’ lives. In many cases, their presence did not simply reinforce a sense of responsibility but acted as a protective factor against inertia and emotional withdrawal, offering participants not only a reason to keep going but also a way to reconnect with life, through touch, care, and companionship. Although these positive emotional transformations were widely described, they were not limited to those who had recently adopted a dog. Both participants reflecting on relatively recent adoptions (within the past year) and those with many years of ownership described similar shifts in emotional stability, comfort, and purpose. This suggests that while the intensity of early adoption may catalyze such changes, the benefits often persisted and became integrated into long-term daily life.

### 3.2. Looking at the Self and the Self Along with Others

#### 3.2.1. Personal Growth and Development

For many participants, living with a dog represented far more than a source of emotional support. Through ongoing interaction and the daily responsibility of care, a space for personal growth emerged, one in which participants experienced meaningful shifts in how they perceived themselves and their relationships with others. Several participants described how the relationship with their dog helped them become more responsible and fostered greater self-awareness. As one participant characteristically noted, “*I’ve become more responsible*, *more emotionally mature*, *more outgoing*”, while another added, “*I’m definitely more responsible now*”, describing an inner shift toward stability and a strengthened sense of duty that stemmed from the consistent need to care for their dog.

This process of caregiving also enhanced skills in emotional regulation and interpersonal expression. Many participants noted that, through living alongside their dogs and the emotional closeness this naturally fostered, they developed a deeper sense of emotional literacy and inner awareness. As reflected in the following quotes, “*I’ve become more patient and more emotionally expressive*”, and “*It’s helped me feel more grateful for the little moments*”, participants conveyed how this connection cultivated new ways of relating to their inner emotional world.

The impact of this relationship, however, did not remain confined to the human–animal bond. Rather, many participants described how the skills nurtured in the context of the dog relationship extended to their interactions with other people. One participant noted, “*With M*, *I learned to observe body language… that really helped me communicate better with other people*”. Increased empathy, attentiveness, and the ability to manage emotions seemed to transfer to wider social interactions, making communication with others more meaningful and authentic.

Within this context, another participant shared how the presence of her dog significantly contributed to her process of socialisation and emotional openness, “*She’s helped me express myself and become more social… I’m quite an introverted person*, *and having T has really helped me communicate better with people*”. This testimony illustrates how coexisting with a dog can facilitate self-expression, boost confidence, and reduce barriers to interpersonal connection. 

Overall, participants’ narratives illustrate how the relationship with a dog can become a subtle yet powerful catalyst for personal growth, fostering self-reflection, emotional literacy, and more authentic connections with others.

#### 3.2.2. Facilitation of Social Interactions

The relationship with the dog did not only serve as emotional support within the participants’ inner world but also opened new pathways for social engagement and interaction with others. Their narratives portrayed the dog as a powerful social catalyst, offering a natural and safe gateway to connection, interaction, and reduced isolation through everyday routines. Several participants described how, after long hours of work, walking their dog served not only as a form of relief but also as an opportunity for communication. As one participant shared, “*I made friends at the park… we walked our dogs*, *they played together*, *and it was nice… I knew that after eight hours at work*, *I’d take her out for a walk and could*, *at the same time*, *meet two or three people there I could talk to*”. This quote illustrates how the dog acted as a “bridge” between the owner and the social environment, offering a predictable and friendly structure for social interaction that might otherwise be absent from the routine of an adult with limited time or few spontaneous opportunities to socialise.

Beyond casual encounters, many participants referred to new connections that emerged unexpectedly through daily walks. “*I still keep in touch with a girl I met at the park… we basically met through our pets*”, one participant recalled, while another added, “*I have a friend I met through our walks at the park… we both have dogs… and now we’re really close*”. These reflections show how an ordinary routine can become the starting point for the development of more meaningful relationships, rooted in shared experience and the natural closeness the dog brings.

The same dynamic was echoed in statements such as, “There have been so many opportunities to meet new people… I’ve interacted with so many at the parks because of A”, “It’s opened doors to new acquaintances”, and “New friendships started through K”. These accounts reveal the role of daily routines with the dog as a mechanism for social reintegration, through which new relationships are formed, enriching the participants’ social lives. The companion dog appeared as a motivating force in initiating connections and even fostering the development of genuine friendships.

In addition, the relationship with the dog seemed to support the maintenance of existing social ties, particularly during times of limited availability. As one participant explained, “*It gives me a reason to get out of the house and go for a walk… and while walking*, *I plan to see people I otherwise wouldn’t have time to meet… my schedule is really tight*”. This reflection demonstrates how the dog’s care routine can provide an opportunity to maintain contact and interaction with meaningful others, even in the context of a demanding lifestyle.

Interestingly, the facilitation of social connections was emphasized across both newer and long-term owners. For those who had adopted their dogs more recently, walking routines often served as an initial gateway into new social circles. For participants with longer ownership histories, these encounters had frequently developed into sustained friendships and ongoing community ties. This indicates that time since adoption may shape the form rather than the existence of such social benefits.

Finally, the dog’s influence extended even to simpler, everyday social expressions, highlighting its role in facilitating interaction with people in ways that may not have occurred otherwise. One participant described, “*In my neighborhood*, *everyone greets me now because of K… before*, *we wouldn’t even say good morning… now we exchange greetings*, *maybe even a few words*”. Even the simplest exchange of a glance or a greeting becomes a meaningful moment of social recognition, reinforcing a sense of belonging and subtly challenging the experience of isolation that many participants reported as part of their daily lives.

Overall, participants’ experiences suggest that the presence of a dog in their lives served not only as emotional support but also as a catalyst for authentic social interaction. While the nature and intensity of these interactions varied among individuals, most agreed on the dog’s role as a bridge to the social world, one that nurtured a sense of companionship and contributed to their broader emotional well-being.

## 4. Discussion

This study explored how dog ownership through adoption influences experiences of loneliness and emotional well-being among adult dog owners. Through rich, qualitative narratives, two major themes were identified: (1) *Alleviating loneliness through companionship* and (2) *Looking at the self and the self along with others*. These themes point to a multifaceted relationship between humans and dogs, one that not only provides emotional comfort but also promotes personal growth and social re-engagement. The findings both support and extend existing research, offering novel insights into the lived experiences of dog owners in a Greek cultural context.

### 4.1. Emotional Shifts and the Role of Companionship

Participants in this study consistently described their adopted dogs as emotionally significant companions who provided comfort, structure, and a sense of presence during periods of emotional vulnerability. This finding aligns with previous research highlighting the psychological benefits of the human–dog bond, particularly its role in emotional regulation, grounding, and mitigating loneliness [12,23,24]. However, unlike studies that examine pet ownership as a general phenomenon, this research suggests that emotional benefits often emerged in the specific context of dog adoption, which was frequently described by participants as a spontaneous and emotionally charged act taken during times of crisis or significant personal distress.

Participants did not depict their dogs as passive sources of comfort but rather as responsive companions whose presence invited mutual attunement, routine, and emotional reciprocity. Their narratives reflected the development of caregiving rituals, daily proximity, and deep emotional reliance—mechanisms that mirror relational frameworks in human–animal studies [21,27]. These interactions appeared to foster stability and emotional relief, particularly in contexts of loneliness or limited human social support, suggesting that the dog–human bond may provide a unique form of affective anchoring.

Supporting this view, prior work has demonstrated that companion animals can play a therapeutic role in reducing anxiety symptoms. For instance, a short walk with a dog yielded greater mental comfort and reduced physiological arousal compared to walking alone [54]. Similarly, longitudinal research has shown that dog ownership is associated with reductions in loneliness among community members, highlighting the potential for dogs to serve as long-term social and emotional buffers [29]. These effects were especially salient during the COVID-19 pandemic, when dogs were widely perceived as sources of connection, routine, and emotional grounding in the face of social isolation [37].

However, these emotional benefits must be interpreted with caution. Emerging research challenges the assumption that pet ownership is universally beneficial. A study examining stress perception among dog owners found that they assessed daily hassles as more stressful than non-owners [61]. Moreover, a stronger human–dog bond was positively correlated with heightened sensitivity to stressful life events [61]. One explanation for this paradox lies in the caregiving responsibilities associated with pet ownership. Dogs may generate feelings of being needed, thereby increasing owners’ sense of responsibility and amplifying the emotional weight of daily stressors. The researchers further argued that public discourse and prior research may have contributed to an overestimation of the dog’s protective role regarding stress, overlooking the potential strain embedded in caregiving obligations [61]. Adding to this complexity, the practical demands of dog ownership extend beyond emotional investment and can include significant economic and time-related costs. For example, research conducted in a European context revealed that owners consistently identify both financial obligations and the time required to care for their dog as meaningful costs, even when they otherwise report a high level of satisfaction with their relationship [62].

This critical perspective is reinforced by findings from a 2023 UK-based study involving individuals with severe mental illness (SMI) [63]. While participants in that study reported high levels of emotional connection with their animals, as measured by the Comfort from Companion Animals Scale, statistical analyses revealed no significant associations between animal ownership and scores related to well-being, depression, anxiety, or loneliness [63]. The perceived strength of the human–animal bond also showed no significant correlation with mental health indicators or type of animal owned [63]. Additionally, a recent quantitative study conducted among Greek male pet owners revealed that although stronger pet attachment was associated with higher levels of perceived social support, it did not translate into measurable reductions in loneliness or improvements in overall psychological well-being [11]. These findings serve as a valuable counterpoint to overly optimistic assumptions in both popular media and scholarship, underscoring that the emotional benefits of pet ownership may be highly context-dependent and not universally applicable.

Taken together, these studies and the present findings suggest that the emotional significance of dog adoption is deeply embedded in relational, situational, and psychological contexts. While some individuals may experience profound emotional shifts and improved well-being through their bond with an adopted dog, others may encounter new stressors, particularly if they lack adequate support or are managing other vulnerabilities. The human–animal bond thus emerges not as a one-size-fits-all remedy, but as a complex relationship shaped by individual histories, emotional needs, and the practical demands of caregiving.

### 4.2. Personal Development and Social Connectedness

Beyond the emotional and psychological benefits frequently associated with dog ownership, participants in this study described how their relationships with their dogs facilitated personal growth and the development of broader social–emotional competencies. The daily demands of caregiving, the emotional closeness fostered through cohabitation, and the routines surrounding dog care were repeatedly described as spaces in which new aspects of the self-emerged, particularly in the domains of self-awareness, responsibility, and emotional regulation.

Participants often attributed a greater sense of personal responsibility, emotional maturity, and self-discipline to their experience of living with a dog. This aligns with research indicating that companion animal ownership can support the development of self-reliance, decision-making, and emotional expression [64,65]. The data further suggest that the structure provided by dog care routines may foster self-management and emotional literacy—skills that fall under the broader category of social–emotional competencies (SECs) [66]. Several participants noted increased patience, greater emotional expressiveness, and a heightened ability to notice and interpret nonverbal cues. These interpersonal skills appeared to transfer beyond the human–animal bond into participants’ interactions with others, enhancing empathy, communication, and emotional availability.

However, these developmental benefits were not uniformly described in abstract psychological terms. Rather, they were situated in the participants’ lived experiences—formed through daily interactions, rituals, and emotionally resonant moments with their dogs. The process of learning to read their dog’s signals or respond to its needs often became a rehearsal ground for more nuanced forms of emotional engagement with others. These findings echo arguments in the literature that dog–human relationships can promote empathy, emotional awareness, and even social perspective-taking [67].

Importantly, this personal growth often occurred in tandem with an expansion of participants’ social worlds. Dogs did not merely support introspective change but also acted as social mediators, creating opportunities for connection that might otherwise remain unavailable, particularly for more introverted or socially isolated individuals. Participants repeatedly described how walking their dogs opened up spontaneous interactions with strangers, created the conditions for forming new friendships, and helped maintain existing social ties in the context of a busy lifestyle. These findings reinforce prior research demonstrating that dogs can function as social catalysts, increasing both the quantity and quality of social interactions [29,68,69]

The socializing effect of dogs is thought to stem from their ability to disrupt conventional norms around public interaction. In one study, participants experienced significantly more community encounters when accompanied by a dog compared to walking alone, suggesting that dogs help normalize interaction across unfamiliar social boundaries [70]. Other studies show that the presence of dogs in communities supports the development of social capital by creating informal networks of reciprocity, friendliness, and civic engagement [71,72]. These informal exchanges—whether greetings on the street, conversations at the park, or shared caregiving tips—are not trivial. They reinforce participants’ sense of belonging and embed them more securely within a community.

Nonetheless, such benefits are not universally distributed. While many participants in the current study reported increased social opportunities through dog walking and care, research has cautioned that the impact of companion animals varies by socioeconomic context and social position. In more affluent neighborhoods, dog ownership tends to correlate with increased social capital and physical activity for both owners and non-owners [69]. However, in lower-income or less socially cohesive areas, women, older adults, and ethnic minorities may experience dogs not as facilitators of sociality, but as barriers—avoiding outdoor spaces out of fear or discomfort [69]. These disparities suggest that while dogs may open doors to social connection for some, they may inadvertently reinforce exclusion or fragmentation for others.

In the present study, however, the reported social benefits of dog ownership—especially in terms of forming new friendships—were clearly valued. These bonds were not superficial but often described as enduring and meaningful, rooted in the shared experiences and routines that emerge through dog ownership. Such connections serve as protective factors against loneliness and foster a deeper sense of relational fulfillment, illustrating how human–dog relationships may act as bridges to broader interpersonal growth and community integration.

### 4.3. Implications and Future Directions

The findings of this study carry important implications for the fields of health, psychology, and education. At the intersection of mental health and social connection, dog ownership emerges as a potentially valuable complementary tool in therapeutic settings, particularly for individuals experiencing chronic loneliness, emotional dysregulation, or psychosocial isolation. Participants described dogs as sources of emotional regulation, routine, and attachment, indicating the potential value of structured human–animal interactions. While the present study focuses on dog ownership through adoption, which involve ongoing caregiving responsibilities and a sustained relational bond, similar benefits may also be achieved in structured settings, such as AAIs, where interaction with non-owned animals occurs in time-limited contexts. This distinction is important, as the nature of the relationship and the conditions of interaction are likely to influence the emotional and psychological outcomes observed. AAIs could therefore be further developed as supportive, low-cost approaches within mental health services, particularly for individuals with limited access to traditional therapy [73,74].

From a public health perspective, the findings suggest that dog adoption, when done responsibly, may serve as both an act of animal welfare and a contributor to individual psychological resilience. Public awareness campaigns and social education programs could incorporate these findings to promote responsible pet adoption not only as a lifestyle choice but also as a strategy for emotional well-being and social reintegration. Community health promotion efforts may also consider the social facilitation role of dogs such as in reducing neighborhood isolation or increasing intergenerational contact in public spaces.

In the field of psychology, especially developmental and health psychology, these findings reinforce the importance of relational factors in emotional development and resilience throughout the life span. Dogs may play a particularly beneficial role during transitional or vulnerable life phases—such as bereavement, post-divorce adjustment, or retirement [74,75,76]—highlighting their relevance in lifespan-focused psychological research and practice.

Additionally, this study holds potential implications for educational and social programs designed to foster emotional literacy, empathy, and caregiving capacity. In educational settings, AAIs or structured dog-based programs may support the development of SECs, particularly among students with emotional or behavioral difficulties. Supporting this, a scoping review has mapped the growing body of international and interdisciplinary research linking HAI to the promotion of SECs in children and youth [66]. The review highlights the emerging nature of this field and offers promising evidence for the effectiveness of universal programs incorporating HAI in enhancing emotional awareness, empathy, and social skills.

Future research should further investigate the long-term psychological impacts of dog adoption across diverse populations and life stages. Longitudinal studies would be particularly valuable in assessing the durability of emotional benefits and how they evolve over time. Research should also examine how factors such as culture, socioeconomic status, living arrangements, and the quality of the human–dog bond mediate these emotional and social outcomes. Finally, given the relational depth described by participants, future inquiries should explore potential risks of emotional overdependence or unrealistic expectations placed on dogs, especially in cases where human social support remains limited.

### 4.4. Strengths and Limitations

This study offers important contributions by examining a Greek sample, a population often underrepresented in the existing literature on companion animal adoption and emotional well-being. The inclusion of participants aged 26 to 72 years enabled the capture of diverse life experiences and perspectives on the psychosocial benefits of dog companionship. The application of qualitative methodology, specifically semi-structured interviews, allowed for a detailed exploration of individual emotional experiences that are often inadequately addressed in quantitative paradigms. Nonetheless, key limitations should be acknowledged. The relatively small and geographically constrained sample limits the generalizability of the findings to broader populations. Furthermore, the predominance of positive narratives among participants may reflect a self-selection bias, wherein individuals with more favorable experiences were more inclined to participate. Additionally, although the inclusion criterion required participants to have adopted their dog at least three months prior, no maximum timeframe was set. As a result, some participants reflected on experiences spanning many years of ownership. This raises the possibility that their accounts capture the cumulative effects of long-term companionship rather than the immediate impacts of adoption itself. Future research should consider setting clearer temporal boundaries or explicitly examining how time since adoption moderates emotional and social outcomes.

## 5. Conclusions

This study adds to the growing evidence that dog ownership through adoption can have a positive impact on emotional well-being and loneliness, particularly when the bond between human and dog is marked by closeness, trust, and mutual responsiveness. The findings reveal that dogs serve not only as companions but also as agents of self-reflection and social reconnection. While the emotional benefits are substantial, they are also nuanced and context-dependent, shaped by individual histories and social environments. By illuminating these experiences through a qualitative lens, this study contributes to a more holistic understanding of the human–animal relationship and its potential role in supporting mental and emotional health.

## Figures and Tables

**Table 1:** Participants’ Characteristics.

Participants	Age	Gender	Occupation	Living Situation	Time Since Dog Adoption (in Months)
DANMEL	26	Female	Student	Lives alone	60
DIMMIL	28	Female	Employed	Lives with partner	38
EIRARI	32	Female	Student	Lives with partner	96
FANIRM	60	Female	Employed	Lives alone	132
GIWSTE	41	Male	Employed	Lives with partner	192
GRIZOU	70	Male	Retired	Lives alone	9
IWACOO	37	Female	Employed	Lives with partner	16
NIKMIK	58	Male	Employed	Lives with family	72
VASART	33	Male	Unemployed	Lives with family	90
KATTZI	21	Male	Employed	Lives alone	96

**Table 2:** Identified Themes and Sub-themes.

Theme	Sub-Theme	Brief Description
Theme 1. Alleviating loneliness through companionship	1. Emotional connection with the dog	Describes the strong emotional bond with the dog, viewed as a family member and source of comfort, stability, and emotional support.
	2. Transformation of well-being	Highlights the improvement in emotional state, including increased positive affect, reduced loneliness, and renewed daily purpose.
Theme 2. Looking at the self and the self along with others	1. Personal growth and development	Reflects increased responsibility, emotional maturity, patience, and enhanced self-awareness through caregiving.
	2. Facilitation of social interactions	Shows how the dog acted as a social catalyst, fostering new friendships and supporting social reintegration.

## Data Availability

The data presented in this study are available on request from the corresponding author due to privacy and ethical restrictions.
